# Fraction of exhaled nitric oxide is higher in liver transplant recipients than in controls from the general population: a cohort study

**DOI:** 10.3389/fimmu.2024.1330923

**Published:** 2024-02-01

**Authors:** Nicoline S. Arentoft, Annette D. Fialla, Paul S. Krohn, Magda T. Patursson, Rebekka F. Thudium, Moises A. Suarez-Zdunek, Julie Høgh, Emilie H. E. Lauridsen, Jesper B. Hansen, Jens-Ulrik S. Jensen, Michael Perch, Dina L. Møller, Hans-Christian Pommergaard, Niels K. Aagaard, Jesper R. Davidsen, Peter Lange, Yunus Çolak, Shoaib Afzal, Børge G. Nordestgaard, Allan Rasmussen, Susanne D. Nielsen

**Affiliations:** ^1^ Department of Infectious Diseases, Copenhagen University Hospital - Rigshospitalet, Copenhagen, Denmark; ^2^ Department of Gastroenterology, Odense University Hospital, Odense, Denmark; ^3^ Department of Surgery and Transplantation, Copenhagen University Hospital – Rigshospitalet, Copenhagen, Denmark; ^4^ Department of Hepatology and Gastroenterology, Aarhus University Hospital, Aarhus, Denmark; ^5^ Department of Gastroenterology, Aalborg University Hospital, Aalborg, Denmark; ^6^ Department of Respiratory Medicine, Copenhagen University Hospital - Herlev and Gentofte, Gentofte, Denmark; ^7^ Department of Cardiology, Heart and Lung Transplant Unit, Copenhagen University Hospital – Rigshospitalet, Copenhagen, Denmark; ^8^ Department of Clinical Medicine, Faculty of Health and Medical Sciences, University of Copenhagen, Copenhagen, Denmark; ^9^ South Danish Center for Interstitial Lung Diseases (SCILS), Department of Respiratory Medicine, Odense University Hospital, Odense, Denmark; ^10^ The Copenhagen General Population Study, Department of Clinical Biochemistry, Copenhagen University Hospital - Herlev and Gentofte, Herlev, Denmark

**Keywords:** liver transplant recipient, fraction of exhaled nitric oxide, pulmonary disease, comorbidity, eosinophilic airway inflammation

## Abstract

**Background:**

Fraction of exhaled nitric oxide with an expiratory flow of 50 mL/s (F_E_NO_50_) is a biomarker of eosinophilic airway inflammation. Liver transplant recipients have an increased risk of pulmonary infections, but little is known about the burden of chronic pulmonary diseases in this group. We aimed to assess the prevalence of elevated F_E_NO_50_ in liver transplant recipients and compare it to controls from the general population.

**Methods:**

F_E_NO_50_ was measured in 271 liver transplant recipients from The Danish Comorbidity in Liver Transplant Recipients (DACOLT) study and 1,018 age- and sex-matched controls from The Copenhagen General Population Study (CGPS). Elevated F_E_NO_50_ was defined as ≥25 or ≥50 parts per billion (ppb). The analyses were adjusted for known and suspected confounders.

**Results:**

The median age of the liver transplant recipients was 55 years (interquartile range (IQR) 46–64), and 58% were men. The liver transplant recipients had a higher median F_E_NO_50_ than the controls [16 ppb (IQR 10–26) vs. 13 ppb (IQR 8–18.), *p* < 0.001]. Furthermore, the liver transplant recipients had a higher prevalence of elevated F_E_NO_50_ (for F_E_NO_50_ ≥25 ppb 27% vs. 11%, *p* < 0.001 and ≥50 ppb 4% vs. 2%, *p* = 0.02). The results were similar after adjusting for age, sex, smoking status, use of airway medication, and blood eosinophil counts [the adjusted odds ratio (OR) for F_E_NO_50_ ≥25 ppb was 3.58 (95% CI: 2.50–5.15, *p* < 0.0001) and the adjusted OR for F_E_NO_50_ ≥50 ppb was 3.14 (95% CI: 1.37–7.20, *p* = 0.007)].

**Conclusion:**

The liver transplant recipients had elevated F_E_NO_50_, implying increased eosinophilic airway inflammation. The clinical impact of this finding needs further investigation.

## 1 Introduction

End-stage liver disease is associated with pulmonary complications, including hepatopulmonary syndrome and portopulmonary hypertension (1, 2). Furthermore, in experimental animal models, liver injury has been shown to cause pulmonary inflammation and tissue damage (3, 4). Liver transplantation is a lifesaving treatment for patients with end-stage liver disease, but pulmonary complications may occur post-transplantation. Thus, liver transplant recipients have an increased risk of pulmonary infections, which can result in long-lasting pulmonary injury that may dispose them to common chronic pulmonary diseases such as chronic obstructive pulmonary disease (COPD) and asthma (5, 6). In addition, liver transplant recipients have an increased risk of drug-induced pulmonary disease due to immunosuppressive medication (7–9). However, little is known about the burden of common chronic pulmonary diseases like COPD and asthma in liver transplant recipients.

Nitric oxide (NO) has important roles in the airways as a vasodilator, bronchodilator, and inflammatory mediator (10). Furthermore, NO is known to be involved in the pathophysiology of some pulmonary diseases (11). NO can be measured in the exhaled breath as the biomarker fraction of exhaled nitric oxide (F_E_NO). Measurement of F_E_NO is recommended in the diagnosis of eosinophilic airway inflammation, and a higher level of F_E_NO is associated with asthma (12, 13). Additionally, F_E_NO is used to assess steroid responsiveness in patients with chronic pulmonary symptoms due to airway inflammation (12).

In the current study, we aimed to assess the prevalence of elevated fraction of exhaled nitric oxide with a flow rate of 50 mL/s (F_E_NO_50_) in liver transplant recipients and in age- and sex- matched controls from the general population and to determine whether liver transplantation is an independent risk factor for elevated F_E_NO_50_. Lastly, we investigate the possible risk factors for elevated F_E_NO_50_ in liver transplant recipients.

## 2 Methods

### 2.1 Study design and population

The Danish Comorbidity in Liver Transplant Recipients (DACOLT) study is a nationwide ongoing non-interventional prospective cohort study that aims to investigate the prevalence, incidence, and pathogenesis of comorbidity in liver transplant recipients (14). All liver transplant recipients aged >20 years who can provide informed consent and who live in Denmark are invited to participate in the study. All liver transplant recipients in Denmark are followed at four regional centers: Copenhagen University Hospital—Rigshospitalet, Aarhus University Hospital, Odense University Hospital, and Aalborg University Hospital. The inclusion of study participants began in April 2021 and is ongoing. The present study includes all participants included in the DACOLT study who had F_E_NO_50_ measured before January 2023.

Age- and sex-matched controls were recruited from the Copenhagen General Population Study (CGPS), an ongoing prospective population-based study since 2003 with >110,000 adult participants aged 20–100 years from the general population around the greater Copenhagen area (15).

The participants in DACOLT and CGPS underwent a physical examination, answered a comprehensive questionnaire on lifestyle, medical history, and medication, and had blood drawn for biochemical analyses, including blood eosinophil count and high-sensitivity CRP (hs-CRP) (14). The protocols for the physical examination and questionnaires were identical for both cohorts. All physically trained healthcare staff performed all physical examinations. Both DACOLT and CGPS have been approved by the Committee on Health Research Ethics of the Capital Region of Denmark (approval number for DACOLT, H-20052199; approval number for CGPS, H-KF-01-114/01). Written informed consent was obtained from all participants. The studies were conducted according to the Declaration of Helsinki.

### 2.2 F_E_NO_50_

F_E_NO_50_ was measured non-invasively using the hand-held NIOX Vero (Aerocrine AB, Sölna, Sweden) device expressed in parts per billion (ppb), with a measurement range of 5–300 ppb (16). The measurements were performed with the participant sitting without a nose clip following the recommendations from the European Respiratory Society and the American Thoracic Society (17). The participants were instructed to inhale to their full lung capacity through the mouthpiece that contained a protective filter to avoid environmental contamination. While exhaling, the participants were guided by an animation on the device to maintain a correct and constant expiratory flow rate of 50 mL/s, with 10% variation allowed. If the participant failed to perform the test correctly, the device automatically required a new measurement.

Elevated F_E_NO_50_ was defined as ≥25 ppb or ≥50 ppb, as recommended by the American Thoracic Society for the interpretation of F_E_NO_50_ in clinical practice (12). F_E_NO_50_ <25 ppb is used as an indication that eosinophilic airway inflammation is less likely, while F_E_NO_50_ ≥50 ppb indicates that eosinophilic airway inflammation is likely (12).

### 2.3 Self-reported variables

Information on asthma, allergy, use of airway medication, and smoking was collected by using a self-reported questionnaire. Asthma was defined as an affirmative response to this question: “Do you have asthma?” Allergy was defined as an affirmative response to this question: “Does food, grass, flowers, or animal hair give you asthma, hay fever, or eczema?” The use of airway medication was defined as an affirmative response to this question “Do you use medication daily or almost daily against asthma/bronchitis (including spray/powder)?”.

Smoking status was defined as either current smoking or current non-smoking, where the latter included both never- and former-smoking individuals. Cumulative smoking was reported in pack-years, defined as the number of years smoking 20 cigarettes per day.

### 2.4 Liver transplantation-related variables

Liver transplantation-related variables were collected from medical records. In the analyses, time since transplantation was defined as 0 to 1 year, 1–5 years, and >5 years post-transplantation. Rejection was defined as ever having had one or more biopsy-verified acute rejections treated with methylprednisolone. The information on the use of immunosuppressive medication at inclusion in the study originated from the national Danish Shared Medication Record (FMK), which includes all medications prescribed in Denmark.

### 2.5 Statistics

Liver transplant recipients from DACOLT were frequency-matched with controls from CGPS on sex and 1-year age intervals, aiming for four controls per liver transplant recipient. The distribution of matching is provided in **Supplementary Tables S1** and **S2**. A *post-hoc* power analysis was conducted, which demonstrated that a study population with 271 liver transplant recipients and 1,018 controls and assuming a 12% prevalence of elevated F_E_NO_50_ ≥25 ppb in the control group (18) would enable us to detect an OR of 1.64 for F_E_NO >25 ppb in the liver transplant group with a power of 80%.

Descriptive statistics were used to compare liver transplant recipients and controls using *t*-tests or Mann–Whitney *U*-test for continuous variables and chi-square tests for categorical variables.

The association between liver transplantation and F_E_NO_50_ was investigated using multiple linear regression. Sex, age, smoking status, self-reported asthma, use of airway medication, and blood eosinophil count were included in a fully adjusted model. Logistic regression was used to investigate the association between elevated F_E_NO_50_ ≥25 ppb and ≥50 ppb, respectively, and liver transplantation using the same adjustment factors as listed above.

We assessed the following pre-defined possible risk factors for elevated F_E_NO_50_: time since transplantation, acute rejection, autoimmune disease as indication for transplantation, use of prednisolone, use of antimetabolites (mycophenolate mofetil (MMF) or azathioprine (AZA) or none of the two), use of calcineurin inhibitors or mTOR inhibitors (tacrolimus or ciclosporin or everolimus or none of the three), elevated high-sensitivity C-reactive-protein (hs-CRP) (≥2.0 mg/L), and elevated eosinophils (≥0.5 × 10^9^/L). The possible risk factors were investigated separately in multiple linear regression adjusted for age, sex, and current smoking status. Risk factors were analyzed in liver transplant recipients only.

We performed three sensitivity analyses: (i) excluding liver transplant recipients with autoimmune disease as indication for transplantation, (ii) including only liver transplant recipients in current treatment with prednisolone, and (iii) excluding liver transplant recipients who were less than 1 year post-transplantation. Furthermore, in exploratory analyses, we investigated elevated interleukin 4 (IL-4), elevated interleukin 6 (IL-6), elevated interleukin 13 (IL-13), and elevated neutrophils (≥5.9 × 10^9^/L) as risk factors for elevated F_E_NO_50_ in liver transplant recipients. Since there is no established consensus on cutoffs for the included interleukins, IL-4, IL-6, and IL-13 were dichotomized with the third quartile as cutoff.

## 3 Results

### 3.1 Study population

We included 271 liver transplant recipients and 1,018 controls. Their characteristics are provided in **Table 1**. The two groups had a comparable distribution of sex with 58% male patients in both groups. The median age was 55 years [interquartile range (IQR): 46–64] in the liver transplant recipients and 57 years (IQR: 48–64) in the controls. The most common indication for liver transplantation was autoimmune disease (45.4%), followed by alcoholic or cryptogenic cirrhosis (22.1%), and the majority of the liver transplant recipients received treatment with a calcineurin inhibitor (91.1%) and MMF (72.0%). The proportions of current smokers and individuals with asthma were comparable between the two groups, while a smaller proportion of the liver transplant recipients had allergies than the controls (24.4% vs. 31.1%).

**Table 1:** Clinical characteristics in liver transplant recipients and controls.

	Liver transplant recipients *n* = 271	Controls *n* = 1,018	*p*-value
Characteristics			
Sex (male), *n* (%)	158 (58.3)	593 (58.3)	1
Age, years, median (IQR)	55 (46–64)	57 (48–64)	0.10
Elevated eosinophils^a^, *n* (%)	7 (2.6)	36 (3.5)	0.57
Smoking and self-reported respiratory morbidity			
Current smokers, *n* (%)	35 (12.9)	142 (13.9)	0.69
Cumulated smoking, pack-years, median (IQR)	9.0 (4.6–20.0)	17.5 (7.5–32)	0.58
Self-reported asthma, *n* (%)	18 (6.6)	74 (7.3)	0.79
Self-reported allergy, *n* (%)	66 (24.4)	317 (31.1)	0.03
Self-reported use of inhalation medicine, *n* (%)	18 (6.6)	60 (5.9)	0.67
Liver transplantation-related variables			
Time since LTX years, median (IQR)	6.8 (2.8–12.2)	NA	
Reason for transplantation^b^		NA	
- Autoimmune liver disease, *n* (%)	123 (45.4)		
o Autoimmune hepatitis	38		
o Primary sclerosing cholangitis	83		
o Primary biliary cholangitis	18		
o Other	8		
- Alcoholic or cryptogenic cirrhosis, *n* (%)	60 (22.1)		
- Cancer, *n* (%)	21 (7.7)		
- Fulminant hepatic failure, *n* (%)	18 (6.6)		
- Metabolic disease, *n* (%)	15 (5.5)		
- Hepatitis C, *n* (%)	11 (4.1)		
- Other, *n* (%)	52 (19.2)		
Immunosuppressive medication at inclusion		NA	
- Prednisolone, *n* (%)	129 (47.6)		
- Tacrolimus, *n* (%)	223 (82.3)		
- Ciclosporin, *n* (%)	26 (9.6)		
- Everolimus, *n* (%)	17 (6.3)		
- MMF, *n* (%)	195 (72.0)		
- Azathioprine, *n* (%)	23 (8.5)		

a Elevated eosinophils defined as counts above 0.5 × 10^9^/L.

b Liver transplant recipients can have more than one reason for transplantation.

NA, not applicable.

### 3.2 F_E_NO_50_

The liver transplant recipients had higher F_E_NO_50_ than the controls [median 16 (IQR: 10–26) vs. 13 (IQR: 8–18), *p* < 0.001]. Compared with the controls, the liver transplant recipients had 1.24 ppb higher F_E_NO_50_ (95% CI: 1.14–1.34, *p* < 0.001) in unadjusted linear analyses and 1.26 ppb higher F_E_NO_50_ (95% CI: 1.17–1.37, *p* < 0.001) when adjusting for age, sex, smoking status, use of airway medication, having asthma, and elevated eosinophils.

### 3.3 Association between liver transplantation and elevated F_E_NO_50_

**Figure 1:**
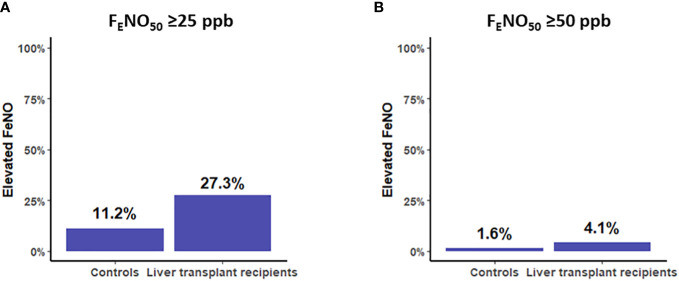
**(A)** Prevalence of the elevated fraction of exhaled nitric oxide (F_E_NO_50_) ≥25 ppb in controls and liver transplant recipients, *p* < 0.001. **(B)** Prevalence of F_E_NO_50_ ≥50 ppb in controls and liver transplant recipients, *p* = 0.02.

**Figure 2:**
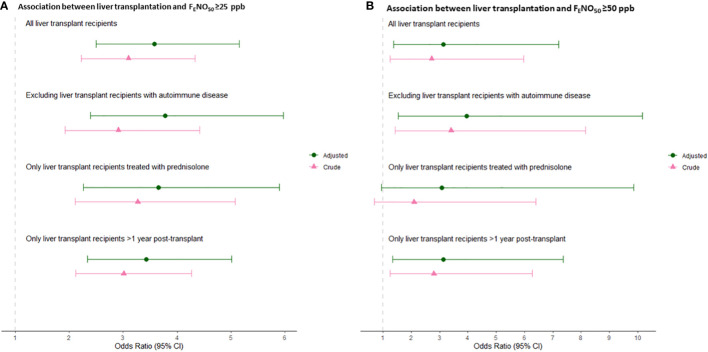
**(A)** Association between liver transplantation and F_E_NO_50_ ≥25 ppb. **(B)** Association between liver transplantation and F_E_NO_50_ ≥50 ppb. Odds ratios and 95% confidence intervals are given for main analyses, including all liver transplant recipients and three sensitivity analyses: (i) excluding liver transplant recipients with autoimmune disease as indication for transplantation, (ii) including only liver transplant recipients treated with prednisolone at the time of inclusion, and (iii) including only liver transplant recipients >1 year post-transplantation.

Elevated F_E_NO_50_ ≥25 ppb was more prevalent in the liver transplant recipients than in the controls [27.3% (95% CI: 21.2%–32.6%) vs. 11.2% (9.3%–13.1%), *p* < 0.001] (**Figure 1A**). Similarly, liver transplantation was associated with a higher risk of F_E_NO_50_ ≥25 ppb, with odds ratios (ORs) of 3.10 (95% CI: 2.22–4.33, *p* < 0.001) in the unadjusted model and 3.58 (95% CI: 2.50–5.15, *p* < 0.001) in the adjusted model (**Figure 2A**). Elevated F_E_NO_50_ ≥50 ppb was likewise more prevalent in the liver transplant recipients than in the controls (4.1% vs. 1.6%, *p* = 0.02) (**Figure 1B**). Liver transplantation was associated with a higher risk of F_E_NO_50_ ≥50 ppb, with ORs of 2.72 (95% CI: 1.25–5.96, *p* = 0.012) and 3.14 (95% CI: 1.37–7.20, *p* = 0.007) in the unadjusted and adjusted models, respectively (**Figure 2B**).

### 3.4 Sensitivity analyses

To test the robustness of the results, we performed a sensitivity analysis excluding liver transplant recipients with autoimmune disease as indication for liver transplantation. Liver transplantation was significantly associated with increased F_E_NO_50_ [1.23 ppb increase (95% CI: 1.12–1.36), *p* < 0.001] as well as with F_E_NO_50_ ≥25 ppb [OR 3.78 (95% CI: 2.39–5.97), *p* < 0.001] and ≥50 ppb [OR 3.95 (95% CI: 1.53–10.17), *p* = 0.004] (**Figures 2A, B**). In addition, we performed a sensitivity analysis including only liver transplant recipients in current treatment with prednisolone, and liver transplantation was still associated with 1.27 ppb F_E_NO_50_ increase (95% CI: 1.15–1.42, *p* < 0.001) and with F_E_NO_50_ ≥25 ppb [OR 3.65 (95% CI: 2.26–5.89), *p* < 0.001] (**Figures 2A, B**). In this analysis, the association between liver transplantation and F_E_NO_50_ ≥50 ppb was similar to the main analyses, albeit with lower power [OR 3.07 (95% CI: 0.95–9.87), *p* = 0.066]. In a sensitivity analysis excluding liver transplant recipients who were less than 1 year post-transplantation, liver transplantation was associated with increased F_E_NO_50_ [1.27 ppb increase (95% CI: 1.17–1.38), *p* < 0.001] as well as with F_E_NO_50_ ≥25 ppb [OR 3.43 (95% CI: 2.34–5.01), *p* < 0.001] and F_E_NO_50_ ≥50 ppb [OR 3.13 (95% CI: 1.33–7.37), *p* = 0.009] (**Figures 2A, B**).

### 3.5 Risk factors associated with higher F_E_NO_50_

**Figure 3:**
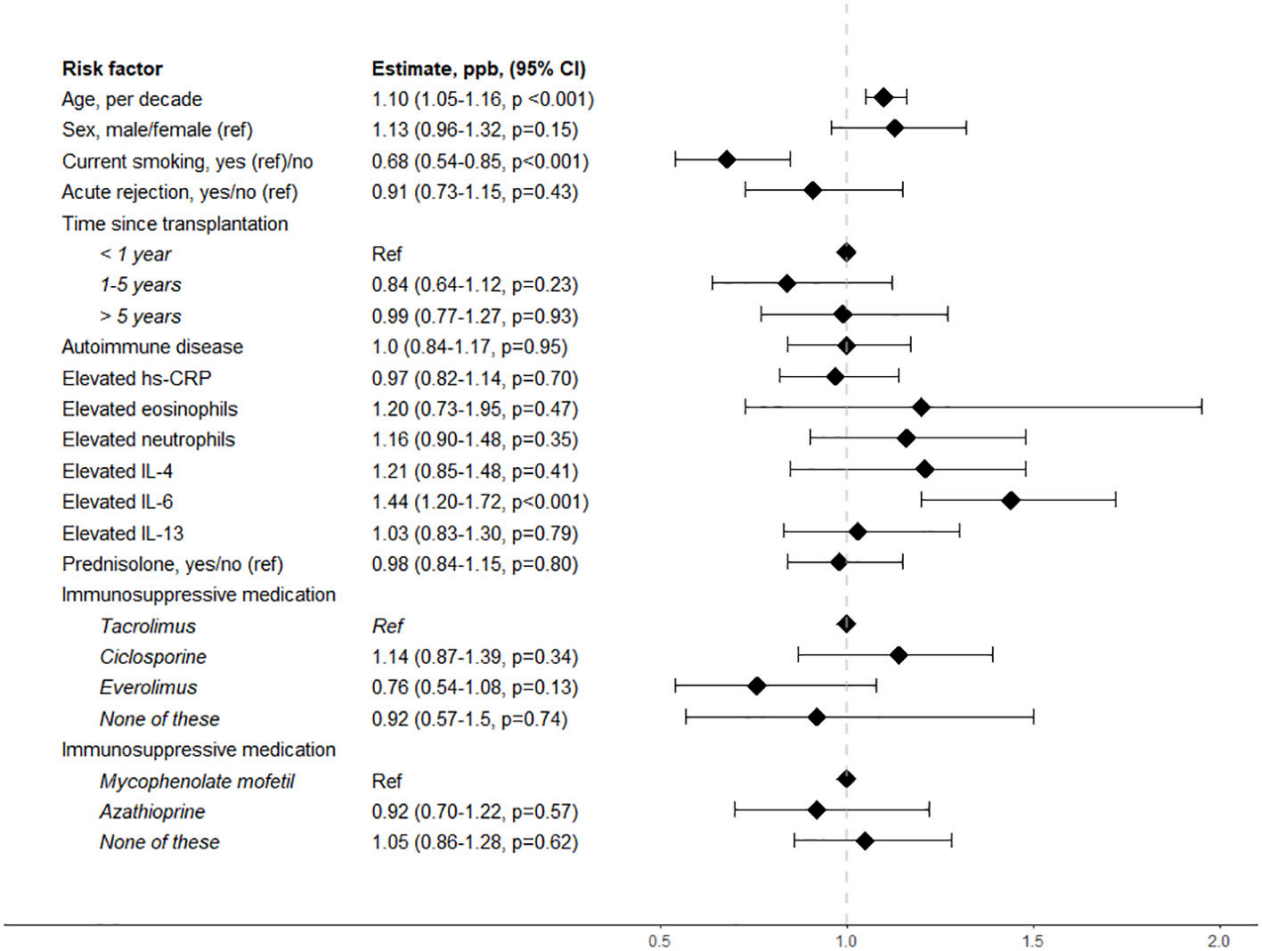
Risk factors associated with F_E_NO_50_ in liver transplant recipients. ref, reference value; hs-CRP, high-sensitivity CRP; IL-4, interleukin 4; IL-6, interleukin 6; IL-13, interleukin 13.

The risk factors associated with higher F_E_NO_50_ in liver transplant recipients included age and current smoking. A 10-year age increase was associated with 1.10 ppb (95% CI: 1.05–1.16, *p* < 0.001) higher F_E_NO_50_. Current smoking was associated with -0.68 ppb (95% CI: -0.85 to -0.54, *p* < 0.001) lower F_E_NO_50_. None of the other tested possible risk factors were significantly associated with F_E_NO_50_ in liver transplant recipients (**Figure 3**).

## 4 Discussion

In this large cross-sectional study of liver transplant recipients and controls from the general population, we found that liver transplantation was significantly associated with higher F_E_NO_50_. Furthermore, liver transplantation was associated with elevated F_E_NO_50_ above clinically relevant cutoffs of 25 ppb and 50 ppb, suggesting that liver transplant recipients have increased eosinophilic airway inflammation. Moreover, the results persisted in sensitivity analyses excluding liver transplant recipients transplanted due to autoimmune disease as well as in analyses including only liver transplant recipients receiving treatment with prednisolone.

Previously, two small studies have investigated exhaled NO in liver transplant recipients. The first study included 18 patients with cirrhosis who underwent liver transplantation and 20 controls (19). Higher F_E_NO was found in patients with cirrhosis prior to transplantation than in controls. There was a decrease in F_E_NO post-transplantation, but the study did not compare F_E_NO in liver transplant recipients with controls (19). In the second study that included 15 controls, 15 patients with cirrhosis, and 30 liver transplant recipients, no differences in F_E_NO could be observed between the groups (20). Both studies were limited by being small and only including selected groups of participants. In addition, they did not have the statistical power to adjust for potential confounders, and their methods of NO measurement were not consistent with the current recommendations. As a result, it is difficult to compare the present study with existing literature. Due to the large number of participants in our cohort with consistent results across sensitivity analyses, we find it likely that F_E_NO is higher in liver transplant recipients. However, validation in an independent cohort is warranted to confirm the present findings.

Measurement of F_E_NO is recommended for the diagnosis of eosinophilic airway inflammation and is often used in the diagnostic work-up of obstructive patients, especially with suspected asthma (12). Thus, higher F_E_NO in liver transplant recipients would be expected if asthma or allergy was more prevalent in this group. In the current study, the prevalence of self-reported asthma was comparable between liver transplant recipients and controls, while allergy was more prevalent in controls. Even though self-reported outcomes are prone to bias, we find it unlikely that asthma or allergy could explain the elevated F_E_NO found in the liver transplant recipients in the present study.

When investigating possible risk factors associated with F_E_NO_50_ in liver transplant recipients, we found that a higher age was associated with higher F_E_NO_50_, while smoking was associated with lower F_E_NO_50_. It has been suggested that smoking decreases F_E_NO_50_, probably due to the downregulation of NO synthase in pulmonary epithelial cells (21). Although factors associated with F_E_NO have been studied in the general population, results have varied (12). Current smoking as a decreasing factor and atopic disease as an increasing factor are often agreed upon, whereas age, sex, height, and ethnicity have shown mixed associations with F_E_NO (12, 22, 23). Since F_E_NO_50_ is used as a marker of airway inflammation, the relation between inflammatory markers and cells and F_E_NO_50_ is interesting. In our analyses, hs-CRP, eosinophils, neutrophils, IL-4, and IL-13 were not associated with F_E_NO_50_, while IL-6 was associated with higher FeNO_50_. IL-4 and IL-13 are anti-inflammatory cytokines known to be involved in nitric oxide production in type 2 asthma in the general population and thus could be expected to be associated with FeNO_50_ in liver transplant recipients (24). In contrast, while IL-6 is also known to be elevated in individuals with asthma, IL-6 is pro-inflammatory and could indicate that systemic inflammation in liver transplant recipients is associated with F_E_NO_50_ (25). The exploratory design of this analysis did not allow for conclusions on the relation between inflammatory markers and FeNO_50_ in liver transplant recipients, but future studies with this focus could be interesting.

Another factor associated with a decrease in F_E_NO is treatment with corticosteroids (26–28). Therefore, measurement of F_E_NO is recommended to assess the likelihood of steroid responsiveness in patients with chronic pulmonary symptoms (12). Almost half of the liver transplant recipients in our cohort were treated with prednisolone at inclusion, but in contrast to our expectations, treatment with prednisolone was not associated with F_E_NO_50_ in our analysis. In a sensitivity analysis, we investigated F_E_NO_50_ in liver transplant recipients treated with prednisolone compared to controls. Surprisingly, in this sub-group, liver transplantation was significantly associated with higher F_E_NO_50_ as well as F_E_NO_50_ >25 ppb. Liver transplantation was only borderline significantly associated with F_E_NO_50_ ≥50 ppb in this analysis, which may be due to a lack of statistical power.

Aside from asthma and allergy, upper respiratory tract infections have also been associated with higher F_E_NO (16, 17, 19, 28, 29). Liver transplant recipients are at an increased risk of respiratory infections due to immunosuppressive medication (7, 8). Respiratory infections are unlikely to be the sole explanation, as F_E_NO only increases during acute infections (29), but respiratory infections often lead to exacerbations in asthma and COPD, possibly contributing to an increase in F_E_NO (30).

Our study had potential limitations. First, information on respiratory morbidity and smoking history was self-reported and recall bias cannot be excluded. Presumably though, recall bias would have affected both liver transplant recipients and controls to the same extent, leading to non-differential misclassification that would bias toward the null hypothesis and would be unlikely to explain our positive findings. Second, information on lung function could have provided information on the clinical impact of higher F_E_NO_50_ in liver transplant recipients. Third, we did not have information on treatment with inhaled corticosteroid for the participants, and we cannot exclude differences in treatment between liver transplant recipients and controls. Our study also had strengths, including the large cohort of liver transplant recipients with age- and sex-matched controls from the general population, examined using identical inclusion protocols. Furthermore, F_E_NO was measured according to the current recommendations, and we adjusted our analyses for possible confounders.

In conclusion, we found liver transplantation to be significantly associated with elevated F_E_NO_50_. This finding suggests that liver transplant recipients have more eosinophilic airway inflammation than the general population. The causality and clinical impact of this finding are unknown, and studies of lung function and underlying pathophysiological mechanisms in liver transplant recipients are warranted.

## Data availability statement

The data that support the findings of this study are available from the corresponding author upon reasonable request.

## Ethics statement

The studies involving humans were approved by Committee on Health Research Ethics of the Capital Region of Denmark. The studies were conducted in accordance with the local legislation and institutional requirements. The participants provided their written informed consent to participate in this study.

## Author contributions

NSA: Conceptualization, Formal analysis, Investigation, Visualization, Writing – original draft. AF: Conceptualization, Project administration, Supervision, Writing – review & editing. PK: Conceptualization, Writing – review & editing. MTP: Conceptualization, Writing – review & editing, Investigation. RT: Conceptualization, Writing – review & editing. MS: Conceptualization, Writing – review & editing, Investigation. JH: Conceptualization, Investigation, Writing – review & editing. EL: Conceptualization, Investigation, Writing – review & editing. JBH: Conceptualization, Writing – review & editing. JJ: Conceptualization, Writing – review & editing. MP: Writing – review & editing, Conceptualization, Supervision. DM: Conceptualization, Writing – review & editing. HP: Conceptualization, Supervision, Writing – review & editing. NKA: Conceptualization, Writing – review & editing. JD: Conceptualization, Writing – review & editing. PL: Conceptualization, Investigation, Writing – review & editing. YÇ: Conceptualization, Investigation, Writing – review & editing. SA: Conceptualization, Writing – review & editing. BN: Conceptualization, Investigation, Writing – review & editing. AR: Conceptualization, Project administration, Supervision, Writing – review & editing. SN: Conceptualization, Funding acquisition, Project administration, Supervision, Writing – review & editing.
