# Supplementary material for: Fraction of exhaled nitric oxide is higher in liver transplant recipients than in controls from the general population: a cohort study
Source: Front Immunol. 2024 Feb 1;15:1330923. doi: 10.3389/fimmu.2024.1330923 (PMC10867152; doi:10.3389/fimmu.2024.1330923)
Supplement: Supplementary file 1 [file Table_1.docx]

**Supplementary material**

**Supplementary Table S1: Distribution of controls for women**

| Age | Number of controls |
| --- | --- |
| 20 | 4 |
| 25 | 2.8 |
| 30 | 2.4 |
| 35 | 3.4 |
| 40 | 4 |
| 45 | 4 |
| 50 | 4 |
| 55 | 4 |
| 60 | 4 |
| >65 | 4 |

**Supplementary Table S2: Distribution of controls for men**

| Age | Number of controls |
| --- | --- |
| 20 | 3.3 |
| 25 | 1.3 |
| 30 | 2.4 |
| 35 | 4 |
| 40 | 4 |
| 45 | 4 |
| 50 | 4 |
| 55 | 4 |
| 60 | 4 |
| >65 | 4 |
